# Columban Simulation Project 2.0: Numerical Competence and Orthographic Processing in Pigeons and Primates

**DOI:** 10.3389/fpsyg.2019.03017

**Published:** 2020-01-17

**Authors:** Damian Scarf, Michael Colombo

**Affiliations:** Department of Psychology, University of Otago, Dunedin, New Zealand

**Keywords:** Null Hypothesis, comparative cognition, numerical competence, orthographic processing, counting, reading

## Abstract

Thirty years ago Burrhus Frederic Skinner and Robert Epstein began what is known as the Columban Simulation Project. With pigeons as their subjects, they simulated a series of studies that purportedly demonstrated insight, self-recognition, and symbolic communication in chimpanzees. In each case, with the appropriate training, they demonstrated that pigeons performed in a comparable manner to chimpanzees. When discussing these studies in the context of his Null Hypothesis, Macphail paid little attention to how the pigeons and chimpanzees solved the tasks and simply assumed that successful performance on the tasks reflected a similar underlying mechanism. Here, following a similar process to the original Columban Simulation Project, we go beyond this success testing and employ the signature testing approach to assess whether pigeons and primates employ a similar mechanism on tasks that tap numerical competence and orthographic processing. Consistent with the Null Hypothesis, pigeons and primates successfully passed novel transfer tests and, critically, displayed comparable cognitive signatures. While these findings demonstrate the absence of a qualitative difference, the time taken to train pigeons on these tasks revealed a clear quantitative difference.

## Introduction

Thirty years ago Burrhus Frederic Skinner and Robert Epstein began what is known as the Columban Simulation Project (Epstein, 1981, 1986, 1991). First floated as the “Pigeon Simulation Project,” “Pigeon” was switched out for “Columban” (derived from the taxonomic name for pigeons) because it sounded more “computer like” (Epstein, 1981). Rather than just a play on words, Skinner and Epstein drew a great deal on the computer simulation literature and their intention was to provide a true simulation, one that “faithfully reproduces all significant characteristics of some phenomenon” (Epstein, 1986, p. 132). What were they trying to simulate? With pigeons as their subjects, they were trying to simulate a series of studies that purportedly demonstrated insight (Kohler, 1925), self-recognition (Gallup, 1970), and symbolic communication (Savage-Rumbaugh et al., 1978) in chimpanzees.

The three studies followed a somewhat similar method (Epstein et al., 1980, 1981, 1984). Pigeons first went through a series of training phases and, following their successful completion, were transferred to the pivotal test that consisted of placing pigeons in a novel situation and observing their behavior. The study of insight provides perhaps the most fruitful example of their approach. The study was based on the work of Kohler (1925), who presented a group of chimpanzees with an intriguing problem. In a large enclosure, Kohler (1925) suspended a banana 2 to 3 m above the ground. Also in the enclosure was a small wooden box. As Kohler (1925) notes, after realizing the banana was out of reach, one chimpanzee “…suddenly stood still in front of the box, seized it, tipped it hastily straight toward the [banana]…began to climb upon it…and springing upward with all his force, tore down the banana” (p. 40–41). Simulating this behavior in pigeons, Epstein et al. (1984) made some basic assumptions about the behaviors that may have led up to the chimpanzee’s behavior. Specifically, they reinforced pigeons to move a small box toward a target and to climb upon the box to reach a small toy banana suspended from the ceiling. In the critical test, they placed the box in one corner and suspended the banana in the other. Mimicking the chimpanzee described above, after initially pacing and looking perplexed, the pigeon pecked/pushed the box toward the banana, stopped underneath it, and then climbed upon the box and pecked the banana.

A discussion regarding whether Kohler’s (1925) chimpanzees and Epstein et al.’s (1984) pigeons truly displayed insight is beyond the scope of the current review. Indeed, Epstein et al. (1981) noted that the concepts themselves, and discussions regarding them, “…impede the search for the controlling variables of the behavior they are said to produce” (p. 696). When discussing these studies in the context of his Null Hypothesis, Macphail (1985) noted that he was “…not concerned here to discuss the nature of the solutions of such problems, whether insight, for example, is a necessary or a useful concept, the key point of interest is the parallel between the chimpanzee and the pigeon performance. There is clearly every reason to suppose that the pigeons solved the problem in exactly the same way as the chimpanzee” (p. 47). While we agree with Macphail’s (1985) first point about the utility, or lack thereof, of concepts such as insight, one could take issue with the second. The point is that similar looking behavior does not imply a similar underlying mechanism and, when one is arguing for the absence of cognitive differences between species, the variables that control the behavior matter. Gallup (1985), when discussing Epstein et al.’s (1981) simulation of his self-recognition study, similarly stated that “Simply because you can mimic the behavior of one species by reinforcing a series of successive approximations to what looks like the same routine in another, it does not follow that the behavior of the former species necessarily arose in the same way” (p. 633).

Mirroring Gallup’s (1985) argument, one could argue that a major limitation of the Columban Simulation Project, and one that may limit its implications for the topics under study, was the focus on what is now termed success-testing (Taylor, 2014). That is, beyond the actual behavior observed (e.g., pecking a blue dot on their body), there were few, if any, additional measures that would allow a closer analysis and comparison of the chimpanzees’ and pigeons’ behavior. An approach that goes beyond mere success-testing is signature-testing, which holds that we “…search for the signatures of various cognitive mechanisms in terms of their errors, biases and limits, rather than a “success-testing” approach where experimenters simply examine whether a problem can be solved or not” (Taylor, 2014, p. 369).

## Columban Simulation Project 2.0

With the aim of testing the limits of Macphail’s (1985) Null Hypothesis, and drawing inspiration from the Columban Simulation Project (Epstein, 1981, 1986, 1991), we set about comparing birds and primates using a signature-testing approach. We initially sought out corvids as experimental subjects. Indeed, work with corvids was rapidly growing at that time and Emery (2006) noted that corvids had displayed abilities that “…are qualitatively and quantitatively more sophisticated than have been demonstrated by other birds, and in many domains comparable to monkeys and apes” (p. 23). Unfortunately, New Zealand is home to only one corvid species (rooks) and their low numbers in the South Island made sourcing the birds extremely difficult. Consequently, much like Epstein (1986) noted when pondering the question of “why pigeons?” we simply went with the materials at hand, and that happened to be the humble pigeon. Constantly on the search for tasks, two high-profile studies presented themselves: first, Brannon and Terrace’s (1998) study on numerical competence and second, Grainger et al.’s (2012) study on orthographic processing. Critically, these studies not only included a novel transfer test (for which we could test for success), but also a number of behavioral metrics that would allow us to compare the signatures/cognitive mechanisms that pigeons and monkeys applied to the tasks.

### Numerical Competence in Monkeys and Pigeons

Numerical competence consists of three concepts, quantity (i.e., cardinality), rank (i.e., ordinality), and counting (i.e., nominal/labeling) (Nieder, 2005). Obviously, in the absence of language, counting is beyond the grasp of non-human animals. Quantity and rank, however, can easily be tested in non-human animals (Chen et al., 1997; Brannon and Terrace, 1998; Scarf and Colombo, 2011; Scarf et al., 2011). Brannon and Terrace’s (1998) study in rhesus monkeys is one of the most powerful examples of cardinality. They trained two monkeys to order stimuli consisting of one, two, three, or four elements. Critically, to ensure the monkey’s behavior was driven by the number of elements in each stimulus rather than other features of the stimuli (e.g., surface area), the elements varied in size, color, and shape. Monkeys were trained on 35 of these 4-item lists and then tested with novel pairs of numerical stimuli. The pairs were one of three types: familiar-familiar (F-F) pairs contained two numerosities drawn from the training range (i.e., 1–4), familiar-novel (F-N) pairs contained one numerosity from the training range (i.e., 1–4) and one numerosity drawn from the novel range of 5 to 9, and novel-novel (N-N) pairs contained two novel numerosities drawn from the 5 to 9 range. Following in Brannon and Terrace’s (1998) footsteps, Scarf et al. (2011) trained four pigeons using an identical paradigm, with the exception that pigeons were trained on 35 3-item, rather than 4-item, lists.

**FIGURE 1:**
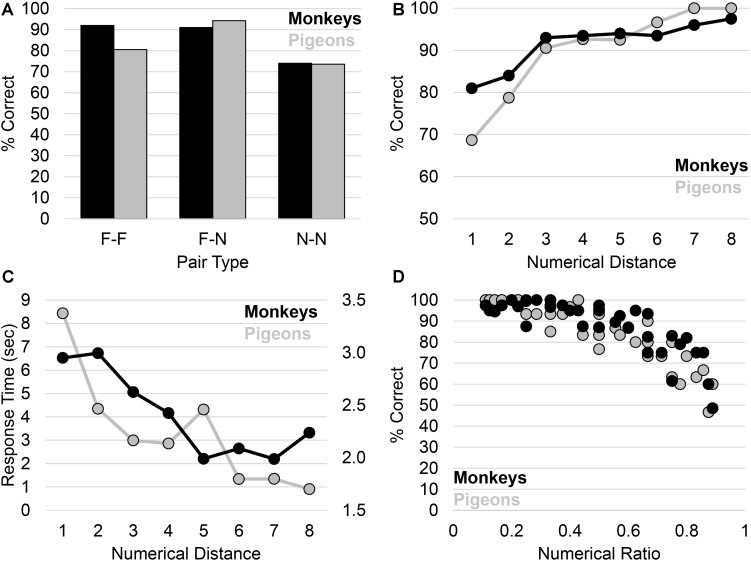
**(A)** Test performance of subjects. **(B)** Test performance of subjects as a function of the numerical distance between the test pair. **(C)** Response time of subjects as a function of the numerical distance between the test pair. **(D)** Test performance of subjects as a function of the numeric ratio between the test pair.

With respect to success testing, consistent with the view that both monkeys and pigeons acquired an abstract numerical rule during training, both performed above chance on the critical N-N pairs (Figure 1A). As one would expect, the monkeys and pigeons also performed well on their respective F-F and F-N pairs (Figure 1A). Following the signature approach, we delved deeper into the behavior of the monkeys and pigeons by assessing two aspects of their performance. First, we assessed the distance effect, the finding that as the distance between two numbers increases, accuracy increases and response time decreases (Moyer and Landauer, 1967; Buckley and Gillman, 1974). For example, subjects should be faster and more accurate with pair 1 vs. 9 (i.e., a distance of 8) than pair 2 vs. 4 (i.e., a distance of 2). Both monkeys and pigeons displayed a clear distance effect, with accuracy increasing (Figure 1B) and response time decreasing (Figure 1C) as the numerical distance between the two stimuli increased.

Second, we investigated whether the performance of the monkeys and pigeons was constrained by Weber’s (1834) law. Weber’s (1834) law reflects the fact that it is not only the distance between stimuli, but also their ratio, that influences discrimination performance. For example, although the distance between pair 1 vs. 2 and pair 8 vs. 9 is 1, the ratio between them is vastly different (0.5 vs. 0.89), thus we would expect performance on pair 1 vs. 2 to be higher than that on pair 8 vs. 9. Consistent with both monkeys and pigeons representing the stimuli in a similar way to humans, their performance was constrained by Weber’s (1834) law in that performance decreased as the numeric ratio increased (Figure 1D).

### Orthographic Processing in Baboons and Pigeons

**FIGURE 2:**
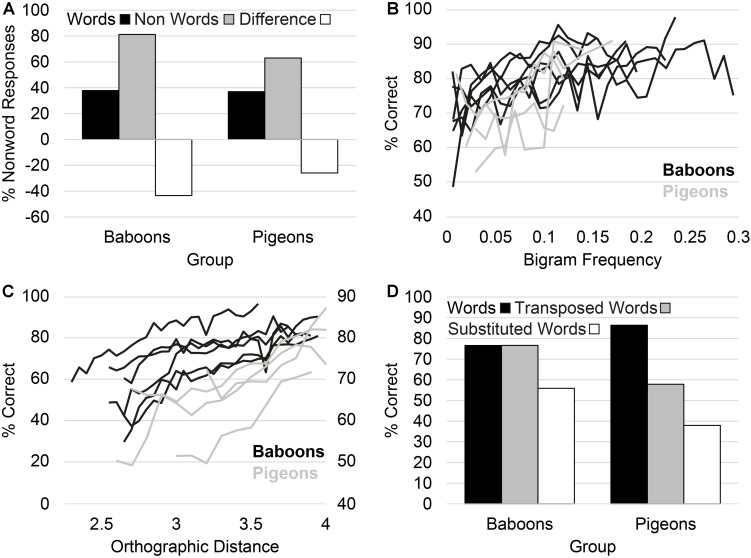
**(A)** Test performance of subjects. **(B)** The performance of subjects as a function of the bigram frequency of words. **(C)** The performance of subjects on non-words as a function of their similarity to words. **(D)** The performance of subjects on the transposed word test.

Learning to read involves the acquisition of letter-sound relationships (i.e., decoding skills) and the ability to visually recognize words (i.e., orthographic knowledge). Much like counting, in the absence of language, decoding skills are human unique. In contrast, recent research and theory suggest that orthographic processing may derive from the exaptation or recycling of visual circuits that are shared by both human and non-human animals (Dehaene, 2009; Dehaene and Cohen, 2011). To test this theory, Grainger et al. (2012) trained six baboons to discriminate four-letter English words (e.g., DONE) from 7,832 four-letter non-words/gibberish (i.e., DMET). Word by word, the baboons acquired vocabularies of between 81 words and 308 words. Following Grainger et al. (2012), Scarf et al. (2016) trained four pigeons using an identical paradigm, with the pigeons acquiring vocabularies between 26 and 58 words. Following training, the success test consisted of presenting subjects with novel words. The baboons and pigeons displayed a similar level of performance with novel words (Figure 2A).

To assess whether the signature underlying their performance matched that displayed by humans, three aspects of the baboons’ and pigeons’ performance was assessed. First, the performance of baboons and pigeons on words increased as the bigram frequency of the words increased (Vinckier et al., 2011). That is, the more frequent certain letter pairs were in the baboons’ and pigeons’ vocabulary, the more accurate they were in responding to them (Figure 2B). Second, the performance of baboons and pigeons on non-words increased as the orthographic similarity between non-words and words in their vocabulary decreased (Figure 2C). Orthographic similarity was measured by calculating each non-words’ Levenshtein (1966) distance, which is the number of changes (e.g., substituting letters in the non-word) required to turn a non-word into a word. For example, to change the non-word DMET into the word DONE, would require substituting letters M, E, and T, for letters, O, N, and E, respectively (i.e., 3 substitutions). Finally, baboons and pigeons were presented with a transposed-letter test (Ziegler et al., 2013; Scarf et al., 2016). The test consisted of presenting subjects with words in which the order of the internal letters were transposed (e.g., “DONE” transposed to “DNOE”), essentially turning them into non-words. Similar to humans, baboons and pigeons showed a tendency to misclassify transposed non-words as words (Figure 2D).

## True Simulations or Circus Trick?

As noted above, Epstein (1981, 1984, 1986, 1991) went to great lengths to explain that the intention of the Columban Simulation Project was to produce true, rather than adequate (i.e., reproduces only some characteristics) or dissimilar (i.e., reproduces no characteristics), simulations (Murphy, 1950). Moreover, Epstein (1986) made clear the simulations were not mere superficial circus tricks, such as a “…circus animal that wears glasses and turns the pages of a book appears to be a reader but does not do these things for the same reasons a person does” (p. 132). An important question is where on this spectrum, from circus trick to true simulation, do the current studies sit? The ability of subjects in both the numerical and orthographic studies to pass novel transfer tests demonstrates that their performance is no surface trick. In fact, we would argue that our simulations are true simulations, and perhaps even stronger simulations than those conducted by Epstein et al. (1980, 1981, 1984). For example, in the studies of numerical competence, monkeys and pigeons displayed two characteristics of human numerical processing, namely the distance effect (Moyer and Landauer, 1967; Buckley and Gillman, 1974) and Weber’s (1834) law. Similarly, in the studies of orthographic processing, baboons and pigeons displayed three features that literate humans display when processing words. Specifically, they perform better on high bigram-frequency words (Grainger et al., 2012), perform better on non-words as their orthographic distance from words increased (Keuleers et al., 2012), and display a clear transposed-letter effect (Perea and Lupker, 2004; Duñabeitia et al., 2014; Tiffin-Richards and Schroeder, 2015).

## Implications for Macphail’s Null Hypothesis

Much like the initial set of studies in the Columban Simulation Project, our work on numerical competence and orthographic processing clearly demonstrates there are no qualitative differences between primates and pigeons on these tasks. Macphail’s (1985, 1987) Null Hypothesis holds that there are also no quantitative differences between species. A quantitative difference is defined as “…one species used a mechanism or mechanisms common to both species more efficiently than the other, and this might be reflected in a faster rate of solution or better asymptotic performance level by one species in some task solved by both” (Macphail, 1985, p. 38). The answer to this question is somewhat more difficult. If our measure of asymptotic performance is based on performance on the novel transfer tests, than the current studies support the Null Hypothesis, with the pigeons performing comparable to the monkeys on the novel numerical pairs (Monkeys: 74% vs. Pigeons: 73.6%) and comparable to the baboons on the novel words (Baboons: 62.1% vs. Pigeons: 63%).

If we use training time as our measure of rate of solution, however, a clear quantitative difference emerges. For example, Brannon and Terrace’s (1998) monkeys acquired their 35 4-item training lists in a matter of months, while Scarf et al.’s (2011) pigeons required well over a year to acquire their much simpler 35 3-item lists. Similarly, Grainger et al.’s (2012) baboons acquired their relatively larger vocabularies (81 to 308 words) in a mere month and a half, while Scarf et al.’s (2016) pigeons took upward of 2 years to acquire their much smaller vocabularies (26 to 58 words). Vast differences in the time required to train pigeons and primates on tasks is something we have observed across an array of tasks (Colombo et al., 2003; Scarf et al., 2018), and supports a clear quantitative difference across animals.

## Conclusion

Macphail (1987) noted that he “…cannot claim strong support for the conclusion that there are no quantitative differences in intelligence” (p. 685). Although alternative training procedures have been shown to drastically impact or reverse differences between animals (Lucon-Xiccato and Bisazza, 2014), based on our extensive experience with pigeons and monkeys, we find it extremely unlikely that any change would eliminate the marked and consistent differences that appear to exist between these groups. While not fulfilling the quantitative component of Macphail’s (1985, 1987) Null Hypothesis, the Columban Simulation Project 2.0 provides convincing evidence that there are no qualitative differences between pigeons and primates on the numerical or orthographic tasks we have studied. Critically, this conclusion holds at both the success and signature level. That is, the absence of qualitative differences holds when we look at the performance of pigeons and primates on the novel transfer tests and, going one step further, look at their respective cognitive signatures (a.k.a., the variables that control the behavior).

## Author Contributions

DS and MC conceptualized and wrote the manuscript.

## Conflict of Interest

The authors declare that the research was conducted in the absence of any commercial or financial relationships that could be construed as a potential conflict of interest.
